# The Responsibility of Silence

**Published:** 1909-11-20

**Authors:** B. Burford Rawlings


*To the Editor of* The Hospital.

### THE RESPONSIBILITY OF SILENCE.

*To the Editor of* The Hospital.

Sir,—My attention has been only recently drawn to the interesting and significant article in your issue of October 16 under the above heading, which ought to be read in connection with another article of the same date concerning "The Re-election of Hospital Staffs."

I doubt if any close observer of hospital work, whose interest is intelligent and apprehensive, will fail in appreciation of the thoughts you have expressed. The medical staff of a hospital, composed as it is of elements usually diverse, sometimes antagonistic, but always disciplined to a display of professional unity in the presence of the layman, presents to the onlooker an undivided front. Yet in the staff's relation to the patients and the investigation and treatment of disease, the public has no such guarantee as a combination of distinguished names appears to afford. Every individual member is a law to himself, and he is as free from articulate criticism as he is safe from interference and supersession. His colleagues may disapprove his methods, but they never allow the fact to be suspected, and if anything untoward happens, he may confidently reckon upon their silence. Were it necessary to enforce this point, some piquant illustrations might be found readily.

So far, I think those familiar with the facts will be agreed. Differences are likely to arise when we seek to propose a remedy. To most people the best course of all is the silence you condemn, and so thoroughly have the staffs succeeded in forcing upon the laity a policy of blind acquiescence that independent judgment in respect of the duty owed by the hospital to the patient has little part with hospital councils.

Obviously, a lay committee unaided cannot decide questions with medical bearings, but what we well may ask is whether it must always remain impossible to erect an advisory medical power distinct from the staff? Of course the staffs would resent the suggestion. Medical exclusiveness has few limitations, and the way to reform is barred by the truly lamentable fact that the large and wholly trustworthy majority of hospital physicians and surgeons would be as strongly opposed to a controlling authority as the small minority whose shortcomings or excesses would be subjected to inquiry and restraint.

Upon another page of the same number of The Hospital where these articles appear, Sir William Church urges that "the school should be entirely separate from the hospital to which it is attached. It should manage its own affairs, independent altogether of the Hospital Board of Governors." This is a subject much too vast to be considered within the compass of a letter, but as not a little of what takes place in the wards is with a view to clinical requirements, and as in some quarters there is a growing disposition to place the school before the hospital, I will suggest shortly that it is desirable both in the interests of the school and as a means of remedying certain evils in the hospital, the school should be brought under the hospital government, and that to this end the government should be virilised by the infusion of a medical element in the person of consultants not charged with active duty in school or hospital.

I am, Sir, your obedient servant,

November 15, 1909. B. Burford Rawlings.

